# Bayesian spatio-temporal modelling of child anemia in Ethiopia using conditional autoregressive model

**DOI:** 10.1038/s41598-022-24475-0

**Published:** 2022-11-24

**Authors:** Zemenu Tadesse Tessema, Getayeneh Antehunegn Tesema, Susannah Ahern, Arul Earnest

**Affiliations:** 1 Department of Epidemiology and Preventive Medicine, School of Public Health and Preventive Medicine, Monash University, Melbourne, Australia; 2 Department of Epidemiology and Biostatistics, Institute of Public Health, College of Medicine and Health Sciences, University of Gondar, Gondar, Ethiopia

**Keywords:** Prognostic markers, Diseases, Nutrition disorders, Malnutrition

## Abstract

Anemia is a common health problem for women and under five children in low income countries. According to the WHO, anemia is considered a serious public health problem when the prevalence is greater than 40%. The prevalence of anemia among children under five in Ethiopia changes over time, and is spatially correlated because it is influenced by environmental, socio-economic and other related factors. However, to our knowledge, there is no small area level estimates of anemia among children under five in Ethiopia. Therefore, this study aimed to assess zonal level estimates of anemia using a Bayesian spatio-temporal conditional autoregressive modeling approach. The data for the study was extracted from the Ethiopian Demographic and Health Surveys (EDHS) from 2005 to 2016. A sample of 18,939 children aged 6–59 months were considered for this study. A Bayesian spatio-temporal conditional autoregressive model was implemented to identify the risk of child anemia. Smoothed relative risks along with the 95% credible interval were reported. The queen’s adjacency matrix method was used in spatial smoothing and in estimating the relative risk. The prevalence of anemia among children aged 6–59 months in Ethiopia was 54% in 2005, 44% in 2011 and 57% in 2016. This study showed that low maternal education, low socio-economic status of women, and maternal anemia at zone level were strongly associated with child anemia in Ethiopia. Therefore, enhancing education for women, improving women's socioeconomic status, and mitigating maternal anemia are crucial to reduce the prevalence of childhood anemia in Ethiopia.

## Introduction

Anemia is a disorder in which there is deficiency of enough healthy red blood cells to carry adequate oxygen to tissues throughout the body^1^. The World Health Organization (WHO) uses the hemoglobin level of less than 11 g/dl as a cut of value to define anemia among children^2^. Nutritional deficiency and malabsorption are the commonest causes of anemia among children under age five^3^. Anemia leads to significant long-and short-term clinical and functional consequences in children such as poor motor and cognitive development, recurrent infections, low school performance and increased risks of comorbidities^4^.

Globally, anemia is a public health problem, and developing countries are highly affected^5–7^. According to WHO, an estimated 1.62 billion people are anemic, in which 43% are observed in low- and middle-income countries (LMICs) particularly in Asian and African countries^8,9^. Under five children and pregnant mothers are highly susceptible segment of community for anemia^1^. Based on a recent EDHS report, the magnitude of anemia among under-five children was 57%^10,11^. Another study which was meta-analysis and systematic review revealed the pooled magnitude of anemia to be 44.83%^11^.

Empirical evidence has been reviewed from the existing literature. The published articles showed that anemia is linked with different socio-economic, environmental and other related factors apart from individual level characteristics. A study done in Debre Markos referral hospital showed that childhood anemia was related with undernourishment, inappropriate complementary feeding initiation time, parasitic infection and family income^12,13^. Other studies showed that anemia among under five children was associated with food insecurity, child age, maternal education, maternal anemia, wealth index and parasitic infection^13–17^.

Previous spatial and multilevel studies using EDHS have been reported in Ethiopia using classical statistical methods and broader regional level geographic boundaries without accounting for spatial correlations^14,16,18,19^. Appropriate small area level estimates (zone level) are needed for effective interventions. In Ethiopia, the zone is the administrative geographic location in which implementation of health service, and operational planning are routinely done. The classical statistical approach, frequentist inference, is based on the likelihood of data to obtain parameter estimates. In addition, frequentist statistics are not intuitive to interpret^20^. This could result in inferential bias since the frequentist approach is totally dependent on the data fit ignoring spatial correlation. Utilizing Bayesian framework can mitigate these limitations. In the Bayesian approach, spatial and spatio-temporal smoothening methods can be fit, which not only considers the effects of the risk factors but also borrows strengths from the neighbouring areas and time periods thereby improving relative risk estimates.

This study aimed to explore the spatio-temporal variation of anemia among under five children using Bayesian spatio-temporal conditional autoregressive models. The Bayesian models allow more interpretable definitions of confidence interval and incorporate prior information for parameter estimation and also include spatial and temporal dependencies via the specifications of prior distribution. Therefore, the main objective of this study was to map and identify risk factors childhood anemia in Ethiopia at zonal level. This study could help zonal health departments to better target specific health service planning, follow up, monitoring and evaluation initiatives.

## Methods and materials

### Study setting

Our study area is Ethiopia, it is an ancient country with a rich diversity of peoples and cultures. Ethiopia is second largest country in Africa with a total population of 120 million^21^. Ethiopia is found at 3° to 14° North and 33° to 44° East with area of 1.1 million square kilometer in East Africa. Ethiopia has 9 reginal and 2 city administrations. The city and regions administrations are subdivided in to 74 zones^22^. So far, a total of four DHSs (2000, 2005, 2011 and 2016) were conducted in Ethiopia.

### Data source

The data were sourced from EDHS conducted in 2005–2016. This study consisted of 18,939 (3296 from the year 2005, 8100 from 2011 and 7543 from 2016) under five children from 74 Ethiopian administrative zones. Spatial merging of enumeration area with zone administrations was performed. The shapefile of Ethiopia was obtained from the website at www.OpenAfrica shapefile at administration level two.

### Sample size and sampling procedure

The DHS program conducted the survey according to following sampling procedures. A total of twenty-one sampling strata have been created and a two-stage sampling technique was employed in each survey 2005, 2011 and 2016. Each region of the country (Amhara, Tigray, Oromia, Afar, Somalia, SNNPRs, Dire-Dawa, Harari, Benishangul-gumuz, Addis Ababa and Gambella) were stratified into urban and rural areas except Addis Ababa (no rural residents available in Addis Ababa) created a total of 21 sampling stratas. In the first stage, 645 EAs in 2016, 624 EAs in 2011, and 540 EAs in 2005 Enumeration Area (EAs) were selected, respectively. An Enumeration Area (EA) holds on average 181 households. In the second stage, a fixed number of households per cluster (EAs) were selected. For this study, the kids record (KR) dataset were used and final sample size were 3296 in EDHS 2005, 8100 in EDHS 2011 and 7543 in EDHS 2016. The detailed sampling procedure is available elsewhere^23–25^.

### Inclusion and exclusion criteria

Under five children who had anemia data in the three survey years 2005, 2011 and 2016 were included. The 2000 survey year was excluded in this study because there was no anemia data in that particular survey year. No similar child was included in each subsequent survey. The survey was conducted every 5 year and they consider different children for each survey.

### Study variables

#### Dependent variable

The number of anemic children (Hemoglobin (Hgb) < 11 g/dl) in each zone (count) was the response variable of the study. Originally, the data was recorded at four level (severe, moderate, mild and non-anemic). The response variable was recoded anemic if a child has a mild, moderate or severe level of anemia, and non-anemic otherwise. Finally, the number of anemic children in each zone was counted and used in the final model analysis.

#### Independent variables

Based on literature^9,26,27^ covariates such as the age group of mother (15–19, 20–24, 25–29, 30–34,35–39, 40–44, and 45–49), residence (urban, rural), region, maternal education (no education, primary, secondary and higher), wealth index (richest, rich, middle, poorest, poorer), maternal anemia status (severe, moderate, mild and non-anemic), sex of child (female, male), birth weight (normal vs underweight) and birth order (1–3, 4–6 and greater that 6) were included in this study.

The proportion of women who had any kind of education in each Zone was calculated and grouped into quartiles. Category one being high proportion education category and category four being low proportion of education category. The proportion of anemic mother in each Zone was calculated and grouped into quartiles. Category one being small proportion of anemic mother and category four being high proportion of anemic mother. The proportion of better socio-economic mothers of each Zone was calculated and grouped into quartiles. Category one being high proportion socio-economic mothers while category four being low proportion socio-economic mother.

#### Risk factors from CAR model

The areal risk factors of child anemia in Ethiopia are presented in the form of relative risk (RR) for both crude and model adjusted smoothed relative risk along with its 95% credible interval.

### Statistical analysis

#### Bayesian spatial–temporal model

Bayesian analysis includes three main components, which are the likelihood (data), the prior distribution and the posterior distribution that represent the combination of the two^28^.

The formulation of Bayesian model is shown below.

The posterior distribution is

1 $$\mathrm{P}\left(\frac{\theta }{}y\right)=\frac{ p(\theta ,y)}{p(y)}= \frac{p(y/\theta )p(\theta )}{p(y)}$$

where $p(y/\theta )$ is the likelihood of y under a model and $p(\theta )$ is the prior density and p(y) is a fixed normalized factor which ensures that the posterior probabilities sum to 1.

Therefore, the posterior distribution is displayed as:

2 $$\mathrm{P}(\theta /\mathrm{y})\propto p(y/\theta ) p(\theta )$$

Based on the above, the posterior distribution is the product of likelihood or data and prior distribution. The main concern in Bayesian analysis is the selection of the prior distribution. In spatial modeling, commonly a non-informative prior or weakly informative prior is used.

For this study, Bayesian spatio-temporal model along with spatially structured and unstructured random effect via conditional autoregressive, normal priors, and linear temporal terms were applied^29^. Model comparison was made based on the Deviance Information Criteria (DIC), and the final model included the model with covariates, structured and unstructured spatial random effects, since it had lowest DIC.

#### Conditional autoregressive model

Conditional autoregressive (CAR) model^29^ is showed below

3 $${\text{O}}_{ik} \sim {\text{Poi}}(\mu_{ik} )$$

4 $${\text{log}}(\mu_{ik} ) = {\text{log}}({\text{E}}_{ik} ) + {\text{u}}_{i} + {\text{v}}_{i + } \beta {1}*{\text{t}}_{k} {\text{ + x}}_{ik}^{\prime } {{\varvec{\upbeta}}}$$

in which O_*ik*_ and E_*ik*_ are the observed and expected anemia count for ith zone (i = 1, 2,…74) and kth time period (k = 1, 2, 3), u_*i*_ is spatially structured random effect and v_*i*_ is spatially unstructured random effect, β1 and β are effect size parameters of time and covariate respectively, x_*ik*_ are the covariates included in the model.

This model included both spatial structured and unstructured random effect parameters in the model for anemia count at zone level in Ethiopia. This modeling approach also considers neighborhood adjacency for estimating the smoothed relative risk, which is an improvement over the crude relative risk.

#### Adjacency matrix

The CAR model uses the adjacency matrix to borrow strength and for parameter estimation. For this study, we created the Queen weight matrix, where each Zone’s immediate neighbors with common points are included. The formulation of adjacency weight matrix is shown below

5 $${\text{w}}_{ik} = \left\{ {\begin{array}{*{20}c} 0 \\ 1 \\ \end{array} \begin{array}{*{20}l} & {otherwise} \\ &{if\;neighbours} \\ \end{array} } \right.$$

where w_*ik*_ weighted matrices *i*th zone (i = 1, 2, 3,…0.74) and *k*th time period (k = 1, 2, 3).

#### Markov Chain Monte Carlo (MCMC)

The estimation obtained from this study was from a Bayesian framework using iterative Markov Chain Monte Carlo (MCMC) sampling techniques, which allows us to estimate the posterior distribution of the model paraments^30^. For this study, Gibbs sampling of MCMC algorithms was used^31^.

#### MCMC convergence

Regarding the convergence in MCMC estimation, the Gelman Rubin statistics and autocorrelation plots were used to ascertain if parameters with 2 different initial values had reached convergence. We used Poisson regression model because there was no evidence of overdispersion (i.e. mean was similar to variance of the outcome). We selected every 10th observation for calculating the posterior distribution to avoid correlated samples. The first 100,000 iterations were discarded (burn-ins) and a further 1000,000 samples were used in the calculations (supplementary file).

#### Model construction

For model construction we used the DIC (deviance information criterion). A model with a lower DIC was considered better. Starting with the model with no covariates, we added 3 variables maternal education, maternal socio-economic status and maternal anemia status. These were determined to be a-priori known risk factors. We found a considerable reduction in DIC from 19,043 to 11,579.

The data management, coding and analysis were done using Stata version 17, WinBUGS, Excel, and ArcGIS 10.7^32^. The summary statistics were obtained from the posterior distribution to describe the covariates and relative risk (RR) with its 95% credible intervals from the Bayesian spatio-temporal conditional autoregressive model. To produce the map, we used the Geographic Information System (ArcGIS v 10.8) software.

### Ethical approval

Ethics approval for this study was obtained from the Monash University Human Research Ethics Committee (Project number 34690).

## Results

### Characteristics of the study population

A total of 18,939 of children aged 6–59 months were included in this study. The non-response rates were 4%, 5% and 5% in EDHS 2005, 2011 and 2016, respectively. A total of 10,393 (54.87%) of children aged 6–59 were anemic from the three surveys. More than half (51.01%) of them were males. The mean (SD) age of mother was 29.45 years (6.6) with the largest proportion (30.59%) being in the 25–29-year age group. In addition, about 16,048 (84.74%) of children were rural residents. More than two-thirds (69.48%) of surveyed mothers had no formal education. Nearly 30% of mothers were anemic, and about 6137 (32.40%) and 3490 (18.43%) of mothers belonged to the poorest and poorer household wealth status, respectively (Table 1).

**Table 1:** Socio-demographic and socio-economic characteristics of child anemia Ethiopia from 2005 to 2016.

Variables	EDHS year			Total (n = 18,939)
	2005 (n = 3,296)	2011 (n = 8,100)	2016 (n = 7,543)	
**Child anemia status**				
Severe	150 (4.55%)	291 (3.59%)	295 (3.91%)	736 (3.89%)
Moderate	943 (28.61%)	2006 (24.77%)	2451 (32.49%)	5400 (28.51%)
Mild	723 (21.94%)	1739 (21.47%)	1795 (23.80%)	4257 (22.48%)
Non-anemic	1480 (44.90%)	4064 (50.17%)	3002 (39.80%)	8546 (45.12%)
**Maternal age group**				
15–19	129 (3.91%)	285 (3.52%)	223 (2.96%)	637 (3.36%)
20–24	617 (18.72%)	1543 (19.05%)	1493 (19.79%)	3653 (19.29%)
25–29	965 (29.28%)	2546 (31.43%)	2282 (30.23%)	5793 (30.59%)
30–34	683 (20.72%)	1719 (21.22%)	1201 (15.92%)	4113 (21.72%)
35–39	547 (16.60%)	1291 (15.94%)	1711 (22.68%)	3039 (16.05%)
40–44	250 (7.28%)	528 (6.52%)	485 (6.43%)	1263 (6.67%)
45–49	105 (3.19%)	188 (3.32%)	148 (1.96%)	441 (2.33%)
**Residence**				
Urban	360 (10.92%)	1232 (15.21%)	1299 (17.22%)	2891 (15.26%)
Rural	2936 (89.08%)	6868 (84.79%)	6244 (82.78%)	16,048 (84.74%)
**Region**				
Tigray	385 (11.68%)	946 (11.68%)	797 (10.57%)	2128 (11.24%)
Afar	162 (4.92%)	768 (9.48%)	725 (9.61%)	1655 (8.74%)
Amhara	442 (13.41%)	912 (11.26%)	741 (9.82%)	2095 (11.06%)
Oromia	702 (21.30%)	1290 (15.93%)	1151 (15.26%)	3143 (16.60%)
Somalia	163 (4.95%)	601 (7.42%)	999 (13.24%)	1763 (9.31%)
Benishangul	255 (7.74%)	711 (8.78%)	649 (8.60%)	1615 (8.53%)
SNNPRs*	633 (19.21%)	1191 (14.70%)	968 (12.83%)	2792 (14.74%)
Gambella	162 (4.92%)	558 (6.89%)	497 (6.59%)	1217 (6.43%)
Harari	156 (4.73%)	401 (4.95%)	367 (4.87%)	924 (4.88%)
Addis Ababa	100 (3.30%)	248 (3.06%)	302 (4.00%)	650 (3.43%)
Dire Dawa	136 (4.13%)	474 (5.85%)	347 (4.60%)	957 (5.05%)
**Maternal education**				
No education	2541 (77.09%)	5721 (70.63%)	4897 (64.92%)	13,159 (69.48%)
Primary	556 (16.87%)	2022 (24.96%)	1927 (25.55%)	4505 (23.79%)
Secondary	182 (5.52%)	239 (2.95%)	479 (6.35%)	900 (4.75%)
Higher	17 (0.52%)	118 (1.46%)	240 (3.18%)	375 (1.98%)
**Wealth index**				
Poorest	856 (25.97%)	2514 (31.04%)	2767 (36.68%)	6137 (32.40%)
Poorer	628 (19.05%)	1521 (18.78%)	1341 (17.78%)	3490 (18.43%)
Middle	645 (19.57%)	1334 (16.47%)	1114 (14.77%)	3093 (16.33%)
Richer	590 (19.90%)	1356 (16.47%)	941 (12.48%)	2887 (15.24%)
Richest	577 (17.51%)	1375 (16.98%)	1380 (18.30%)	3332 (17.59%)
**Sex of child**				
Male	1657 (50.27%)	4134 (51.04%)	3869 (51.29%)	9660 (51.01%)
Female	1639 (49.73%)	3966 (48.96%)	3674 (48.71%)	9279 (48.99%)
**Birth weight**				
≤ 2.5 kg	2400 (72.82%)	5607 (69.22%)	5501 (72.93%)	13,508 (71.32%)
> 2.5 kg	896 (27.18%)	2493 (30.78%)	2042 (27.07%)	5431 (28.68%)
**Birth order**				
1–3	1551 (47.06%)	4084 (50.42%)	3870 (51.31%)	9505 (50.19%)
4–6	1069 (32.43%)	2608 (32.20%)	2437 (32.31%)	6114 (32.28%)
> 6	676 (20.51%)	1408 (17.38%)	1236 (16.39%)	3320 (17.53%)
**Maternal anemia status**				
Severe	48 (1.46%)	84 (1.04%)	121 (1.63%)	253 (1.34%)
Moderate	304 (9.22%)	485 (5.99%)	730 (9.81%)	1519 (8.06%)
Mild	652 (19.78%)	1341 (16.56%)	1758 (23.63%)	3751 (19.92%)
Non-anemic	2292 (69.54%)	6191 (76.42%)	4830 (64.93%)	13,312 (70.68%)

*EDHS Ethiopian Demographic and Health surveys, *SNNPRs* Southern Nation, Nationalities and Peoples.

### Risk factors from CAR model

The Bayesian spatio-temporal conditional autoregressive model was fitted to identify risk factors of child anemia in Ethiopia at the areal level. From the results, education, economic status and maternal anemia were associated with child anemia. Better maternal education level was associated with lower child anemia. Mothers living in education level category 3 zones had a 16% increased risk of having an anemic child than mothers in category 1 (RR = 1.16, 95% CrI 1.09–1.34); and mothers in education category 4 had a 12% increased risk of having an anemic child than mothers in category 1 (RR = 1.12, 95% CrI 1.02–1.18). Maternal socio-economic status had an impact on child anemia in Ethiopia. Mothers living in category 3 and 4 zones had a 17% and 15% increased risk respectively of having an anemic child than women in socio-economic category 1 (RR = 1.17, 95% CrI 1.09–1.25) and (RR = 1.15, 95% CrI 1.06–1.24), respectively. Maternal anemia status is associated with child anemia in Ethiopia. Mothers anemic status under category 2 had a 18% increase of having an anemic child than category 1 (RR = 1.18, 95% CrI 1.05–1.27). In addition, anemic mother who are under category 3 had 23% increase of having anemic child than category 1 (RR = 1.23, 95% CrI 1.12–1.36) (Table 2).

**Table 2:** Fixed effect parameter estimation relative risk (RR) and its 95% credible interval for Bayesian spatio-temporal Autoregressive model result.

Variable	Category	Mean (95% CrI)	SD	RR* (95% CrI)
Alpha (intercept)		− 0.094 (− 0.198, 0.006)	0.050	0.91 (0.82, 1.01)
Time		− 0.0032 (− 0.028, 0.021)	0.012	0.99 (0.97, 1.02)
Category of low maternal education	Quartile1 (high education level)	Ref		Ref
	Quartile 2	− 0.012 (− 0.0798, 0.0519)	0.032	0.98 (0.92, 1.05)
	Quartile 3	0.149 (0.01, 0.301)	0.031	1.16 (1, 01, 1.35)*
	Quartile4 (low education level)	0.114 (0.025, 0.17)	0.032	1.12 (1.02, 1.18)*
Category of low socio-economic status	Quartile 1 (better economic status)	Ref		Ref
	Quartile 2	0.064 (− 0.006, 0.1374)	0.037	1.05 (0.99, 1.14)
	Quartile 3	0.158 (0.090, 0.2267)	0.034	1.17 (1.09, 1.25)*
	Quartile 4 (low economic status)	0.144 (0.066, 0.2216)	0.039	1.15 (1.06, 1.24)*
Category maternal anemia	Quartile 1 (low maternal anemia)	Ref		Ref
	Quartile 2	0.168 (0.051, 0.241)	0.039	1.18 (1.05, 1.27)*
	Quartile 3	0.213 (0.122, 0.311)	0.038	1.23 (1.12, 1.36)*
	Quartile 4 (high maternal anemia)	0.065 (− 0.0072, 0.1461)	0.041	1.06 (0.99, 1.15)

*Significant at alpha 5%; *RR* relative risk, *SD* standard deviation.

### Convergence of the model

Convergence was seen after 100,000 iterations from BGR statistics plot, autocorrelations plots and MC error less than 5% of SD, indicating all the diagnostic tests were fulfilled the convergence criteria (supplementary file one).

### The spatial and temporal distribution crude relative risk anemia in Ethiopia

#### Crude relative risk of anemia in Ethiopia

**Figure 1:**
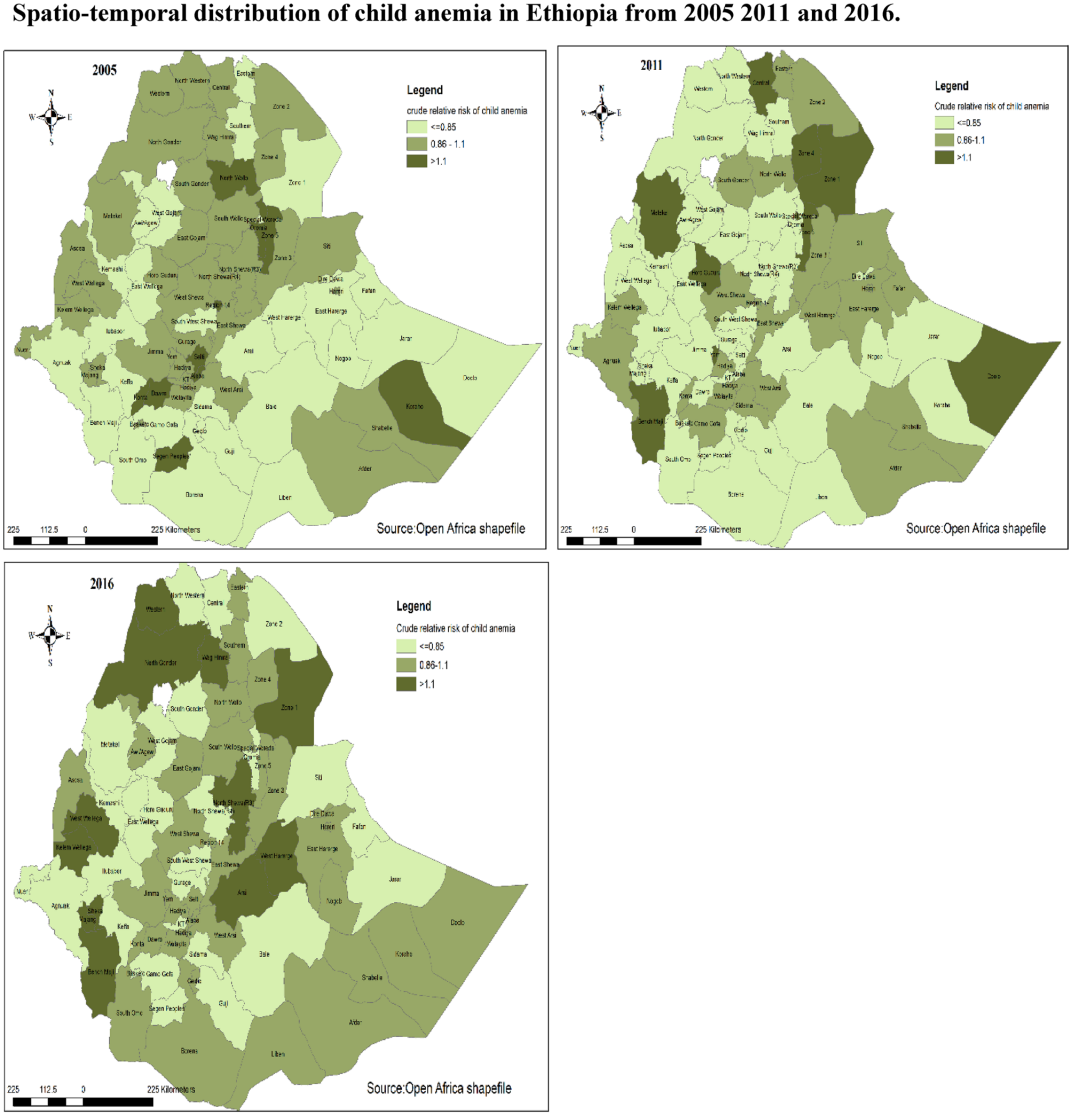
Crude relative risk of anemia in Ethiopia from 2005 to 2016 (source: https://africaopendata.org/dataset/ethiopia-shapefiles).

The crude relative risk of anemia map was generated without considering any spatial correlation or smoothing. The observed number of child anemia and direct standardization was used to obtain the expected count of child anemia for each zone. By using observed and expected counts of child anemia the crude relative risks were generated. The higher crude relative risks of anemia from most recent time point (2016) were located in western Afder (RR = 1.51), zone one (RR = 1.53) and South Gondar (RR = 2.23). The lower crude relative risks were located in gurage (RR = 0.64), Nuer (RR = 0.35) and selti (RR = 0.26). The white color in the map is lake tana which is unpopulated^32^ (Fig. 1) (Appendix 1).

#### Smoothed relative risk of anemia in Ethiopia

**Figure 2:**
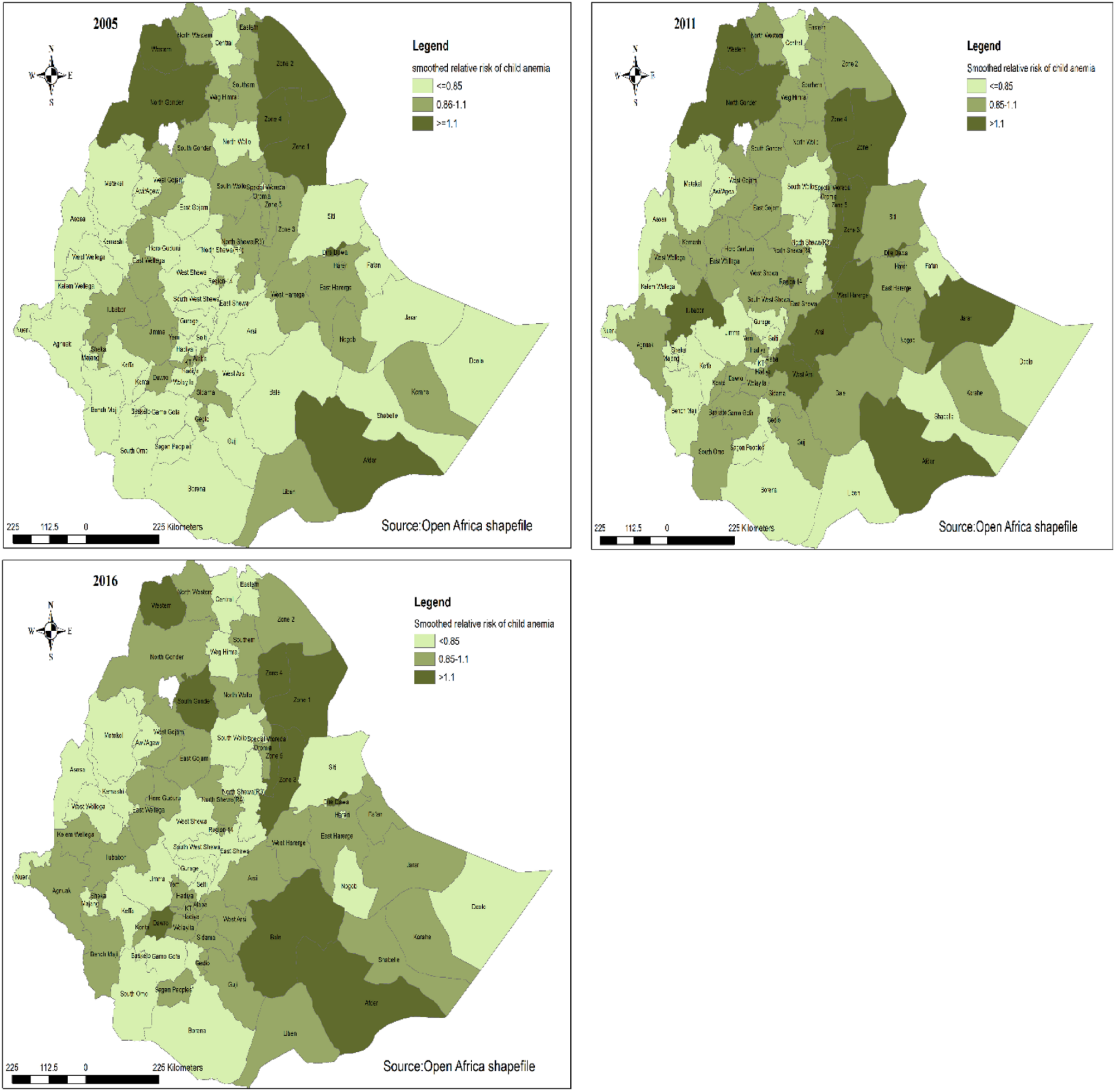
Smoothed relative risk of anemia in Ethiopia from 2005 to 2016 (source: https://africaopendata.org/dataset/ethiopia-shapefiles).

The smoothed relative risk of anemia in Ethiopia for the three consecutive survey years (2005, 2011 and 2016) were mapped and shown below. The map has three classifications of smoothed relative risk (RR < 0.85, 0.8–1.1 and > 1.1) for each survey year for ease of comparison across the three time periods. The darker color indicates a higher risk of having anemic children and the lighter color indicates a lower risk of having anemia as compared to the other zones. The white color in the map is lake tana which is unpopulated^32^ (Fig. 2) (Appendix1).

#### Posterior probability of being anemic in Ethiopia

**Figure 3:**
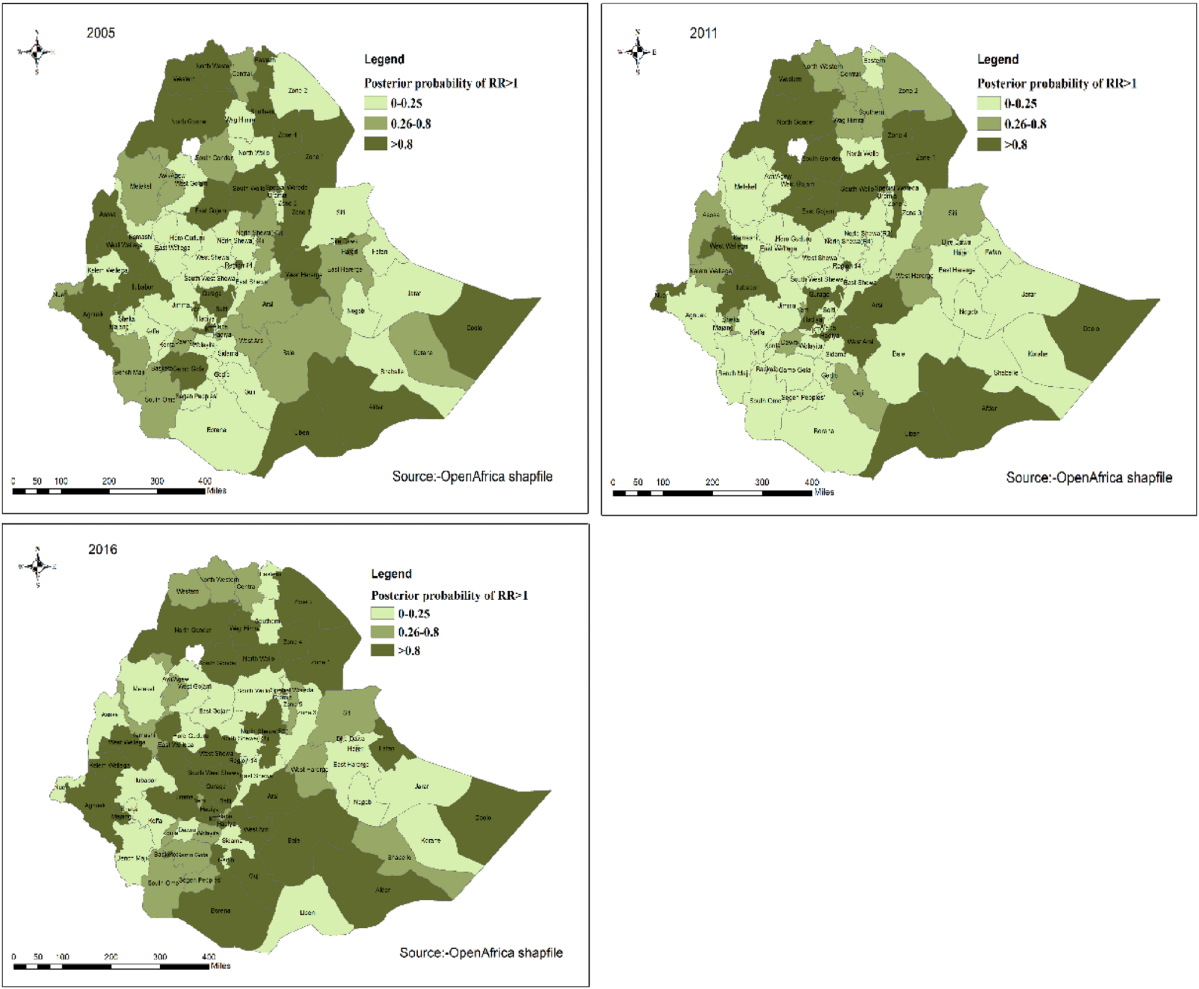
Posterior probability of anemia in Ethiopia from 2005 to 2016 (source: https://africaopendata.org/dataset/ethiopia-shapefiles).

The posterior probability of having anemic children in Ethiopia from 2005 to 2016 were mapped and displayed below. The darker color indicates areas that had high probability of having anemic children (posterior probability > 0.8). The lighter color indicates areas that had low probability of having anemic children (posterior probability 0–0.25). The white color in the map is lake tana which is unpopulated^32^ (Fig. 3) (Appendix1).

## Discussion

The prevalence of anemia among under-five children in Ethiopia was 54% in 2005, 44% in 2011 and 57% in 2016^33^. Based on the WHO criteria, anemia prevalence greater than 40% are categorized as a severe public health problem. The Ethiopian data from 2005 to 2016 confirmed that anemia among under-five children in Ethiopia was a major public health problem throughout this period. The empirical evidence by both multilevel and spatial analysis using classical statistics showed that studies done at 9 regions and two city administrations was a global estimate and therefore is difficult to design effective interventions at Zone level. Small area level estimate at zone level and robust statistical method of analysis is crucial to mitigate the prevalence of anemia among under five children. The Bayesian spatio-temporal conditional autoregressive model are needed for mapping risk and to identify risk factors for this severe public health problem in Ethiopia. Therefore, for this study, a Bayesian spatio-temporal conditional autoregressive model was applied to obtain small area estimation by considering adjacency matrix to find smoothed relative risk and it 95% credible interval.

The Bayesian conditional autoregressive spatio-temporal model result showed that zone-level maternal education, socio-economic status and maternal anemia status were associated with child anemia in Ethiopia.

The study revealed that maternal education was significantly associated with child anemia. Women with low category of maternal education had increased risk of having anemic children compared to women who had a higher education level. This finding was consistent with studies reported in Korea^11,34^. This could be due to the fact that maternal education is directly linked to family wealth status and child nutrition. Children born to mothers with low education level may not get adequate nutrition such as iron rich food like meat and poultry due to poor affordability of food items and lack of knowledge about iron-rich foods for children. In addition, uneducated mothers were less likely to utilize child health services which may have a significant and positive impact on child health^14^.

This study evidenced that maternal socio-economic status or wealth index had a strong link with child anemia. As the wealth index category of women becomes poorer, there is a high likelihood of having anemic children as compared to women with better socio-economic status. This finding was consistent with studies done in sub-Saharan Africa^35,36^, Namibia^37^ Guinea^38^, Uganda^39^ and India^40^. Ethiopia is a poorly developed country and its people rely on seasonal farming. The country also faces different droughts due to El Nino episodes, where the rainfall is below normal and this contributes for lack of food in many parts the country. Alternatively, women in a better socio-economic category have greater ability to feed her children iron rich food^41^.

Another significant risk factors of childhood anemia in this study was maternal anemia status. As the level of anemia status of women category increases, there is a high likelihood of having an anemic child as compared to non-anemic women. This finding is in line with studies from in Ethiopia^14^, India^41^, Southern Africa^42^. The possible reason might be mothers and their children will have similar exposure to infections and other environmental factors that affect red blood cells production and iron storage^42^. In addition, children born from anemic mothers are more likely to have low level of essential minerals intake like zinc, iron and folate as well as vitamin A and B12^14^.

Finally, the map of smoothed relative risk of childhood anemia in Ethiopia showed that there is variation of childhood anemia distribution in Ethiopia across zones as indicated in Fig. 1. Future researchers can utilize this baseline data as a potential case control study by comparing high child anemic zones (case) and non-affected zones (controls) through a targeted case–control study, to better understand particular local factors related to the higher burden of childhood anemia.

In addition, for this mapping we used Queens method of adjacency with an order one. There are many types of adjacency for mapping such us Queens method of adjacency with order two and above, block weights, Rook contiguity and the like^43^. Therefore, future research can evaluate the impact of different adjacencies on the results.

### Strength and weakness of the study

This study has some strengths. First, it is national representative data and it is generalizable. Second, it is unique method of analysis not done before in the literature having local level estimate using Bayesian spatio-temporal modeling using adjacency matrix which a robust method. This study’s findings will help healthcare administrators to design effective spatial based interventions to reduce childhood anemia in the country, and to establish local case control studies with high anemic area as case and low anemic area as control to show causal effect.

This study has its own limitations. First, since the data is collected at a point in time it is not possible to show causal effect relationships. Second, some important variable possible affect childhood anemia like malaria, HIV/AIDS, hookworm, human development index, gross domestic product, and population size are not recorded in the dataset. Third, the data is not the most recent as the 2021 survey was not conducted due to civil war and COVID-19.

## Conclusion

The prevalence of child anemia in Ethiopia is 54% in 2005, 44% in 2011 and 57% in 2016. Based on WHO criteria, an anemia prevalence greater than 40% is categorized as a severe public health problem. This study used a new methodology to confirm that low maternal education at the zone-level, low socio-economic status of a women and maternal anemia is strongly linked with child anemia in Ethiopia. Therefore, promoting the education of women, enhancing the socio-economic status of women, and reducing maternal anemia by implementing spatial based strategies are highly recommended to alleviate childhood anemia in Ethiopia. In addition, geographic targeted intervention case–control studies can further generate a hypothesis on possible explanations for the disparity we have found geographically.

## Supplementary Information

Supplementary Information 1.

Supplementary Information 2.

## Data Availability

The datasets used during the current study are publicly available data from measure DHS program found at www.dhsprogram.com.

## Abbreviations

CAR: Conditional autoregressive
CrI: Credible interval
DHS: Demography and health survey
EDHS: Ethiopia demography and health survey
MCMC: Markov Chain Monte Carlo
RR: Relative risk
SNNPR: Southern nation nationalities of people region
WHO: World Health Organization
