# Supplementary material for: Bayesian spatio-temporal modelling of child anemia in Ethiopia using conditional autoregressive model
Source: Sci Rep. 2022 Nov 24;12:20297. doi: 10.1038/s41598-022-24475-0 (PMC9700834; doi:10.1038/s41598-022-24475-0)
Supplement: Supplementary file 1 — Supplementary Information 1. [file 41598_2022_24475_MOESM1_ESM.docx]

**Supplementary files**

**Diagnostic plots**

**Gelman Rubin (BGR) Statistics plot for Markov chain Monte Carlo convergence check**

**supplementary figures 1: -** **Gelman Rubin (BGR) Statistics plot**

**Autocorrelation plot for Markov chain Monte Carlo convergence check**

**supplementary figures 2:- Autocorrelation plot**
